# Dual Phases of Respiration Chain Defect-Augmented mROS-Mediated mCa^**2+**^ Stress during Oxidative Insult in Normal and **ρ** ^0^ RBA1 Astrocytes

**DOI:** 10.1155/2013/159567

**Published:** 2013-03-10

**Authors:** Tsung-I Peng, Muh-Shi Lin, Mei-Jie Jou

**Affiliations:** ^1^Department of Neurology, Keelung Medical Center, Chang Gung Memorial Hospital, Keelung, Taiwan; ^2^Department of Medicine, Chang Gung University, Tao-Yuan, Taiwan; ^3^Department of Surgery, Faculty of Medicine, School of Medicine, National Yang-Ming University, Taipei, Taiwan; ^4^Department of Neurosurgery, Taipei City Hospital, Zhongxiao Branch, Taipei, Taiwan; ^5^Department of Physiology and Pharmacology, College of Medicine, Chang Gung University, 259 Wen-Hwa 1st Road, Kwei-Shan, Tao-Yuan 333, Taiwan

## Abstract

Mitochondrial respiratory chain (RC) deficits, resulting in augmented mitochondrial ROS (mROS) generation, underlie pathogenesis of astrocytes. However, mtDNA-depleted cells (**ρ** ^0^) lacking RC have been reported to be either sensitive or resistant to apoptosis. In this study, we sought to determine the effects of RC-enhanced mitochondrial stress following oxidative insult. Using noninvasive fluorescence probe-coupled laser scanning imaging microscopy, the ability to resist oxidative stress and levels of mROS formation and mitochondrial calcium (mCa^2+^) were compared between two different astrocyte cell lines, control and **ρ** ^0^ astrocytes, over time upon oxidative stress. Our results showed that the cytoplasmic membrane becomes permeated with YO-PRO-1 dye at 150 and 130 minutes in RBA-1 and **ρ** ^0^ astrocytes, respectively. In contrast to RBA-1, 30 minutes after 20 mM H_2_O_2_ exposure, **ρ** ^0^ astrocytes formed marked plasma membrane blebs, lost the ability to retain Mito-R, and showed condensation of nuclei. Importantly, H_2_O_2_-induced ROS and accompanied mCa^2+^ elevation in control showed higher levels than **ρ** ^0^ at early time point but vice versa at late time point. Our findings underscore dual phase of RC-defective cells harboring less mitochondrial stress due to low RC activity during short-term oxidative stress but augmented mROS-mediated mCa^2+^ stress during severe oxidative insult.

## 1. Introduction 

Astrocytes are the most abundant type of glial cells that provide support and nutrition for neurons in the central nervous system (CNS). A crosstalk between astrocytes and neurons is crucial to maintain CNS homeostasis. Numerous neurologic disorders are generated due to the disturbance of the interactions between astrocytes and neurons, such as cerebral ischemia, neurodegeneration, cerebral edema, and hepatic encephalopathy [1]. Astrocyte-mediated neuroprotection has been proposed to be due to the maintenance of energy metabolism, adjusting osmolarity for volume regulation [2], the control of synaptic transmission and neurovascular coupling [3–7], and the limiting of neuronal death from excitotoxins and oxidants such as glutamate and reactive oxygen species (ROS) [8, 9]. Crucially, astrocytes enhance neuronal antioxidant defense by releasing high intracellular levels of antioxidants to the extracellular fluid around neurons [10]. 

The mitochondrial respiratory chain (RC) is crucial for cell survival due to its primary role in ATP generation. In physiological condition, mitochondrial ROS (mROS) are generated by the RC during ATP synthesis due to the leakage of electrons primarily from complex I, complex III and more recently complex II to molecular oxygen (O_2_) [11–13]. However, augmented mROS formation is evident in RC defects [14]. Molecular pathogenesis of astrocytes can be initiated and enhanced by mitochondrial oxidative stress, which is due to augmented mROS generation. Mitochondrial oxidative stress leads not only to the interruption of the energy supply but also to severe damage of mitochondrial components including proteins, lipids, and mitochondrial DNA (mtDNA). In addition, oxidative stress is often accompanied by mitochondrial calcium (mCa^2+^) overload, which leads to mROS increase [15]. Subsequently, these stresses act synergistically for a vicious amplification of additional oxidative stress which leads to the activation of mitochondrial permeability transition- (MPT-) dependent and/or MPT-independent release of mitochondrial lethal proteins including cytochrome c from the intermitochondrial space; these changes culminate in the “point of no return” apoptosis [16]. Thus, RC injuries could be one of the critical mechanisms impairing astrocyte-mediated neuroprotection. 

*ρ* ^0^ cells are defined as those lacking mitochondrial genome, encoding for 2 rRNAs, 22 tRNAs, and 13 protein subunits that form RC complexes I, III, IV, and V. Therefore, *ρ* ^0^ cell line is an *in vitro* model to investigate the role of RC in CNS injuries and diseases. Controversy exists over the opposite capacity of *ρ* ^0^ cells to exert protective effects: they are sensitive [17, 18] or resistant [19, 20] to apoptosis. Since RC is a pivotal component of cell death, its role in apoptotic regulation is warranted to be well understood. 

In the current study, we sought to assess the effects of RC-enhanced mitochondrial stress in astrocytes following oxidative insult. For this purpose, we have compared the ability to resist oxidative stress between the two different astrocyte cell lines, normal (control) and *ρ* ^0^ astrocytes, over time. Our results showed that *ρ* ^0^ astrocytes displayed moderate level of mROS and mCa^2+^ surg under minor oxidative stress but augmented formation of mROS and mCa^2+^ during severe oxidative insult. 

## 2. Materials and Methods

### 2.1. Cell Preparation

Normal rat brain astrocytes (RBA-1) used for this study were originally established through a continuous passage of primary astrocytes isolated from 3-day-old JAR-2, F51 rat brains by Dr. Jou et al. [21]. All cells were grown in Dulbecco's modified Eagle's medium (Life Technologies, Grand Island, NY, USA) supplemented with 10% (v/v) fetal bovine serum. The cells were plated onto glass coverslips (Model no. 1, VWR Scientific, San Francisco, CA, USA) coated with poly-L-lysine. Experiments were preformed after cells grew to 80–90% (about 2-3 culture days) of confluence. The mtDNA-free cells, *ρ* ^0^, were obtained from human 143B osteosarcoma cells treated with ethidium bromide (100 *μ*g/mL). *ρ* ^0^ cells were maintained in Dulbecco's modified Eagle's medium containing 10% fetal bovine serum supplemented with high glucose (4.5 g/mL), pyruvate (0.11 mg/mL), and uridine (0.1 mg/mL). 

### 2.2. Chemicals and Fluorescent Dyes Loading for Studying Mitochondrial Function

All chemicals were obtained from Sigma (St. Louis, MO, USA) and fluorescent dyes were purchased from Molecular Probes Inc. (Eugene, OR, USA). Mitochondrial function was studied by imaging mitochondrial morphology, mROS formation, mitochondrial membrane potential (DeltaPsi(m)) changes, mCa^2+^ regulation, and the opening of the MPT using specific fluorescent probes. To image mitochondrial morphology, cells were loaded with a mitochondrial-targeted fluorescent probe, Mito-Tracker Green (Mito-G, giving a green fluorescence) or Mito-Tracker Red (Mito-R, giving a red fluorescence), both at a concentration of 100 nM. DeltaPsi(m) was detected using either 200 nM and the ratiometric indicator 5,5′,6,6′-tetra-chloro-1,1′,3,3′-tetraethylbenzimidazolylcarbocyanine iodide (JC-1) or 300 nM tetramethyl rhodamine ethyl ester (TMRM). Intracellular ROS formation and mROS were visualized using the acetyl ester form of 2 *μ*Mdichlorofluorescein (DCF-DA) and 100 nM dihydrorhodamine 123 (D-123), respectively. mCa^2+^ was detected using 1 *μ*M Rhod-2/AM. Fluorescent probes were all loaded at room temperature for 20–30 min. After loading, cells were rinsed three times with HEPES-buffered saline (in mM : 140 NaCl, 5 KCl, 1 MgCl_2_, 2 CaCl_2_, 10 glucose, 5 HEPES, and pH 7.4). For all ester forms of dyes including DCF-DA and Rhod-2/AM, cells were treated for an extra 40 min after dye loading to allow cleavage of the ester form of dye to its acid form.

### 2.3. Fluorescence Conventional and Multiphoton Imaging Microscopy

All phase-contrast and conventional fluorescence images were obtained using a Zeiss inverted microscope (Axiovert 200M, Carl Zeiss, Jena, Germany) equipped with a mercury lamp (HBO 103), a cool CCD camera (CoolSNAP fx, Roper Scientific, Tucson, AZ, USA), and Zeiss objectives (Plan NeoFluar 100, NA 1.3 oil). Filters used for detecting DCF were no. 10 (Exi: BP 450–490 nm; Emi: BP 515–565 nm) and for Mito-R and TMRM the filter was no. 15 (Exi: BP 546/12 nm; Emi: LP 590 nm). Confocal images of cells and mitochondria were collected on a Bio-Rad Radiance 2100 using 488 nm Argon laser illumination (Bio-Rad, Hercules, CA, USA). To avoid single-photon-induced autolysis of DCF/DA, multiphoton illumination was applied as previously described [22]. Multiphoton fluorescence images were collected on a Leica SP2 MP (Leica-Microsystems, Mannheim, Germany) fiber-coupling system equipped with a Ti:Sa-Laser system (model: Millennia/Tsunami; Spectra-Physics) providing a pulse repetition rate at 82 MHz, laser pulse width of 1.2 picoseconds, a spectral bandwidth of 1 nm, and object pulse width of 1.3 picoseconds. Wavelength at 800 nm with an average laser power of 600 mW was selected for illumination. During fluorescence imaging, the illumination light was reduced to the minimal level by using appropriate neutral density filters to prevent the photosensitizing effects due to the interaction of light with fluorescent probes including bleaching and autooxidation of the fluorescent probes. All images were processed and analyzed using MetaMorph software (Universal Imaging Corp., West Chester, PA, USA). Intensity levels were analyzed from the original images and graphed using Microsoft EXCEL software and Photoshop. Pseudocolor display with a scale ranged from 0 to 255 units was used to enhance the contrast of the fluorescence changes for each image.

### 2.4. Quantification of ROS Generation and mCa^2+^ Using a Fluorescent Spectrofluorimeter

The cells cultured in 96-well standard black/clear bottom plate (Greiner Bio-One International AG, Kremsmünster, Austria). The ROS was detected with DCF (excitation and emission wavelengths were 490 and 520 nm, resp.). mCa^2+^ was detected with Rhod-2 (excitation and emission wavelengths were 561 and long pass 575 nm, resp.). The fluorescent intensity of DCF and mCa^2+^ were quantified on a Spectramax Gemini EM spectrofluorimeter (Molecular Devices, Sunnyvale, CA, USA). Results were analyzed by using SoftMax Pro 4.7 software (Molecular Devices) and Microsoft Excel software.

### 2.5. Measurement of Cell Viability

Cell viability was detected using the colorimetric 3-(4,5-dimethyl-2-thiazolyl)-2,5-diphenyl-2H-tetrazolium bromide (MTT) assay as previously described [23]. Activity of a mitochondrial reductase to convert a soluble tetrazolium salt into an insoluble formazan precipitate was measured by an ELISA reader (A-5082; TECAN, Grödig/Salzburg, Austria). To investigate the protective effects of RC-defective (*ρ* ^0^) astrocytes on hydrogen peroxide- (H_2_O_2_-) induced cytotoxicity, control and *ρ* ^0^ astrocytes were treated with different concentrations of H_2_O_2_ for different periods of time. MTT assay was performed after 1 hr of H_2_O_2_ exposure. Activity of mitochondrial reductase was calculated as the amount of MTT dye conversion relative to the changes seen in sham-treated control cells. Data were represented as mean ± SE of at least three independent experiments. 

### 2.6. Measurement of Cellular Oxygen Consumption

Cells (5 × 10^5^/100 *μ*L) after trypsinization were immediately transferred to the chamber of Mitocell equipped with a Clark oxygen electrode (782 Oxygen Meter; Strathkelvin Instruments, Glasgow, UK) for the measurement of cellular O_2_ consumption as previously described [24]. The rate of O_2_ consumption was calculated from the slopes and expressed in % of O_2_ consumed per min per 5 × 10^5^ cells.

### 2.7. Statistical Analysis

The results were expressed as mean ± SEM, and statistical significance was evaluated by either one-way or multifactorial analysis of variance (ANOVA). A value of *P* < 0.05 was considered significant. Each experiment was repeated at least three times.

## 3. Results

### 3.1. Slower O_2_ Consumption Rate in *ρ* ^0^ RBA1 Astrocytes

**Figure 1:**
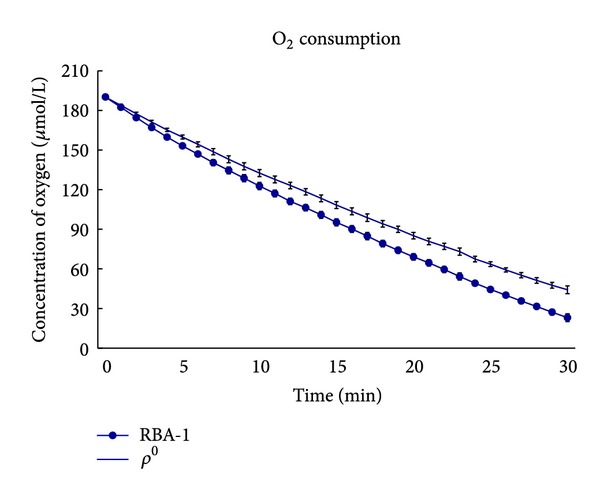
*ρ* ^0^ (RC-lacking) RBA1 astrocytes exhibited lower RC activities. RC activities are indicated by O_2_ consumption and ATP production. In comparison, *ρ* ^0^ astrocytes consumed O_2_ slower than control cells. Moreover, O_2_ consumption compromised significantly during long-term oxidative stress in *ρ* ^0^ astrocytes.

RC activities are presented with O_2_ consumption and ATP production [25, 26]. Therefore, we further examined whether a difference exists in O_2_ consumption between control and *ρ* ^0^ (RC-lacking) RBA1 astrocytes. The result showed that control cells consumed oxygen faster than *ρ* ^0^ astrocytes (Figure 1). However, O_2_ consumption compromised significantly during long-term oxidative stress in *ρ* ^0^ astrocytes. These findings indicate relatively low level of RC activity, leading to less mitochondrial stress in *ρ* ^0^ astrocytes, but the protective effect would be abolished over a long time interval. 

### 3.2. Dual Phases of *ρ* ^0^ RBA1-Astrocytes-Mediated Protection

**Figure 2:**
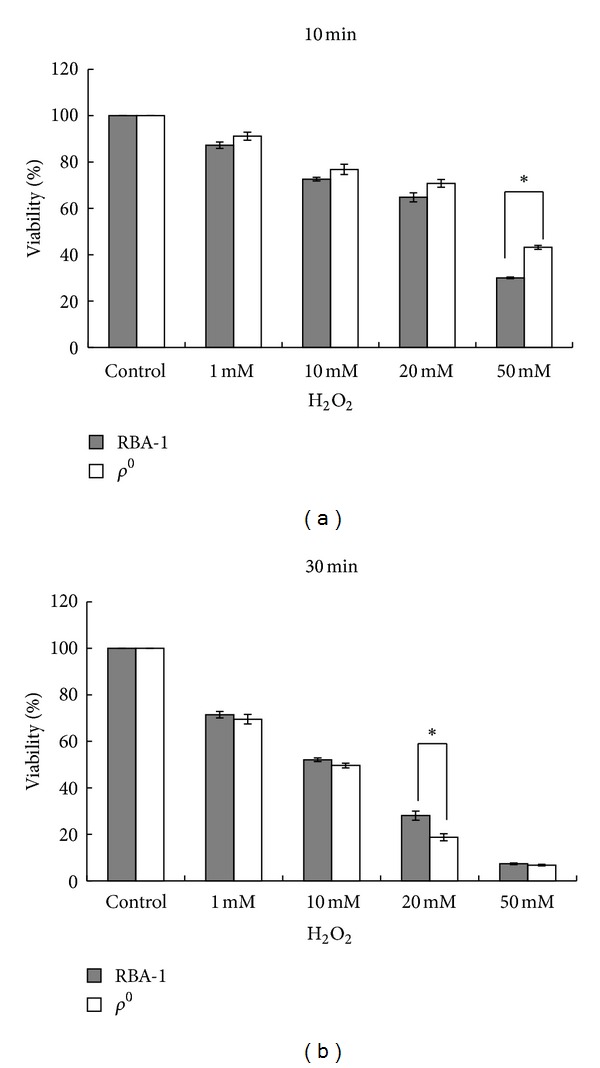
RC defect-augmented loss in cell viability during H_2_O_2_ stress as measured by the MTT assay. (a, b) Dose-dependent cytotoxic effect induced by H_2_O_2_ exposure in control (filled columns) and *ρ* ^0^ RBA1 astrocytes (empty columns); (a) time point for H_2_O_2_ exposure: 10 min and (b) 30 min. There were no statistically significant differences between the two groups in the cell viability at different concentration for 10 minutes (*P* > 0.05) (a). 20 mM H_2_O_2_ caused a statistically significant loss in cell viability in *ρ* ^0^ astrocytes for 30 min when compared to control cells (*P* < 0.001) (b). Data were expressed as mean values ± SE of four separate experiments. *P < 0.001.

The extent of oxidative stress depends on toxin concentration and exposure time [27, 28]. To determine the optimal concentration and exposure time, control and *ρ* ^0^ RBA1 astrocytes were treated with different concentrations of H_2_O_2_ (0, 1, 10, 20, and 50 mM) for different periods of time (10, 30 min) to establish an oxidative stress model. Cell viability following H_2_O_2_ exposure was measured by the MTT assay in the two groups. H_2_O_2_ (1–50 mM) significantly decreased the cell viability of both cells in a concentration-dependent manner. There were no statistically significant differences between the two groups in the cell viability at different concentration for 10 minutes (*P* > 0.05) (Figure 2(a)). As shown in Figure 2(b), the high concentration of H_2_O_2_ (50 mM) caused a significant loss in cell viability. We did not use this extremely high dose of H_2_O_2_ because it was likely that a high concentration would have been excitotoxic to astrocytes. Furthermore, 20 mM H_2_O_2_ caused a statistically significant loss in cell viability in *ρ* ^0^ astrocytes for 30 min when compared to control cells. Thus, optimal H_2_O_2_ concentration of 20 mM was determined for further experiments. 

### 3.3. RC-Defective Astrocytes Augmented Mitochondrial Damage during Oxidative-Stress-Induced Apoptosis

**Figure 3:**
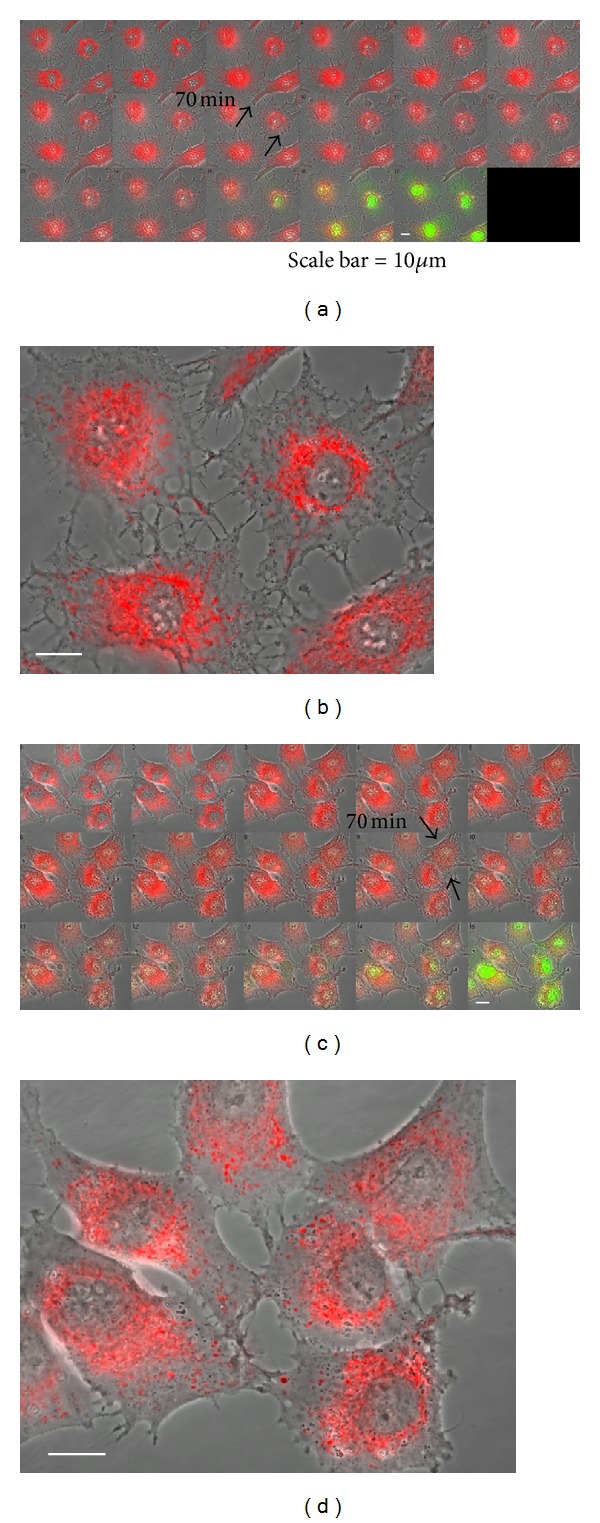
RC-defective astrocytes augmented mitochondrial damage during oxidative stress-induced apoptosis. (a, b) RBA1 astrocytes and (c, d) *ρ* ^0^ RBA1 astrocytes. Time-lapse fluorescence images demonstrate dynamic changes in mitochondrial morphology using Mito-R (labeled in red and apoptotic nuclei using YO-PRO-1 (labeled in green color) during H_2_O_2_ stress. The first image of each image series in (a), (c) is the control. Dual fluorescence time-lapse images of Mito-R and YO-PRO-1 were taken simultaneously at 10 min interval for 150 min. (b), (d) Show magnification 400x of time-lapse fluorescence images at 70 mins in (a), (c), respectively. Scale bar =10 *μ*m.

**Figure 4:**
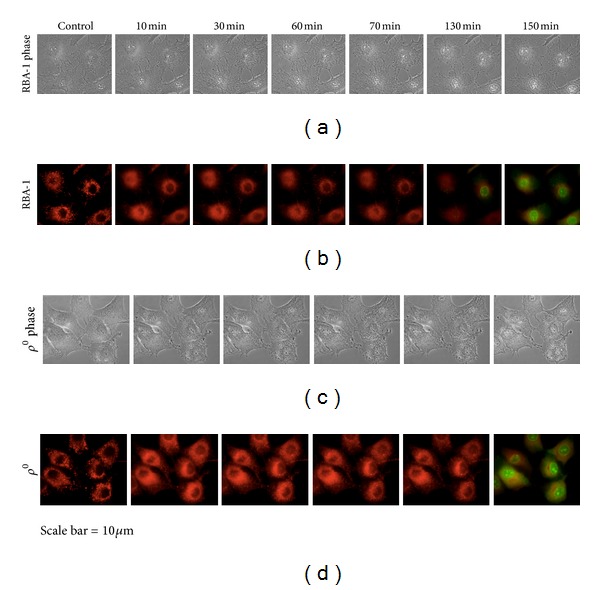
Time-lapse fluorescence images at selected time points. (b) Six time-lapse Mito-R, YO-PRO-1 images of normal RBA1 astrocytes. RBA-1 astrocytes become permanent to YO-PRO-1 after treatment with 20 mM H_2_O_2_ at 150 min. (d) Five time-lapse Mito-R, YO-PRO-1 images of *ρ* ^0^ RBA1 astrocytes. *ρ* ^0^ RBA-1 astrocytes become permanent to YO-PRO-1 after treatment with 20 mM H_2_O_2_ at 130 min. (a) Phase-contrast images of normal RBA1 astrocytes in six time-lapse series. (c) Phase-contrast images of *ρ* ^0^ RBA1 astrocytes in five time-lapse series. Images were taken at rest and after exposure of H_2_O_2_ at 0, 10, 30, 60, 70, 130, and 150 min. Bar =10 *μ*m.

To compare the capacity to resist to long-term oxidative stress between control and *ρ* ^0^ astrocytes, we used time-lapse measurements and recorded the detailed changes in cellular as well as mitochondrial morphology during the entire apoptotic cell death process induced by 20 mM H_2_O_2_ using both conventional phase-contrast and confocal fluorescence imaging microscopy coupled with a noninvasive mitochondria-targeted fluorescent probe, Mito-R and fluorescent apoptosis marker, YO-PRO-1.  Figure 3 shows time-lapse fluorescence images showing dynamic changes in mitochondrial morphology (labeled in red) and apoptotic nuclei (labeled in green) during H_2_O_2_ stress in control (a, b) and *ρ* ^0^ RBA1 astrocytes (c, d). In Figures 3(a) and 3(c), first image of each image series is the control. Dual fluorescence time-lapse images of Mito-R (for morphology) and YO-PRO-1 (for apoptotic nuclei labeling) were taken simultaneously at 10 min interval for 150 min. For analysis, simultaneous visualization of mitochondrial morphology labeled with Mito-R and apoptotic nuclei labeled with YO-PRO-1 were compared between the two groups over 6 time points: 10, 30, 60, 70, 130, and 150 min in Figure 4. Apoptotic events including swelling of mitochondria, plasma membrane blebbing, YO-PRO-1 stain, and nuclear condensation [29, 30] of the two groups were summarized in Table 1. In comparison, cellular morphology of both groups after 20 mM H_2_O_2_ stress which showed phase dense gray mitochondria in the cytosolic area became much lighter due to the swelling of mitochondria over time. Under laser scanning confocal imaging microscopy, mitochondria began to swell earlier on exposure to H_2_O_2_ in *ρ* ^0^ astrocytes than control group (10 min versus 20 min, resp.). There was no significant difference between the two groups for plasma membrane blebbing. Figures 3(b) and 3(d) show magnification 400x of time-lapse fluorescence images at 70 mins in Figures 3(a) and 3(c), respectively. In comparison, *ρ* ^0^ astrocytes formed more marked plasma membrane blebs than in control group over time. Moreover, time to become YO-PRO-1-positive was earlier in *ρ* ^0^ astrocytes than control group (120 min versus 140 min, resp.). In addition, the percentage for decreased nuclear sizes prior to and after H_2_O_2_ treatment is much higher in *ρ* ^0^ astrocytes than in control group (8.9% versus 7.2%, resp.). These results strongly suggest that *ρ* ^0^ astrocytes lacking RC exert protection only in minor oxidative stress while become much vulnerable to secondary oxidative insults in severe oxidative stress. 

### 3.4. Dual-Phase Alteration in mROS and mROS-Dependent mCa^2+^ Formation upon Oxidative Stress Extent in *ρ* ^0^ Astrocytes

**Figure 5:**
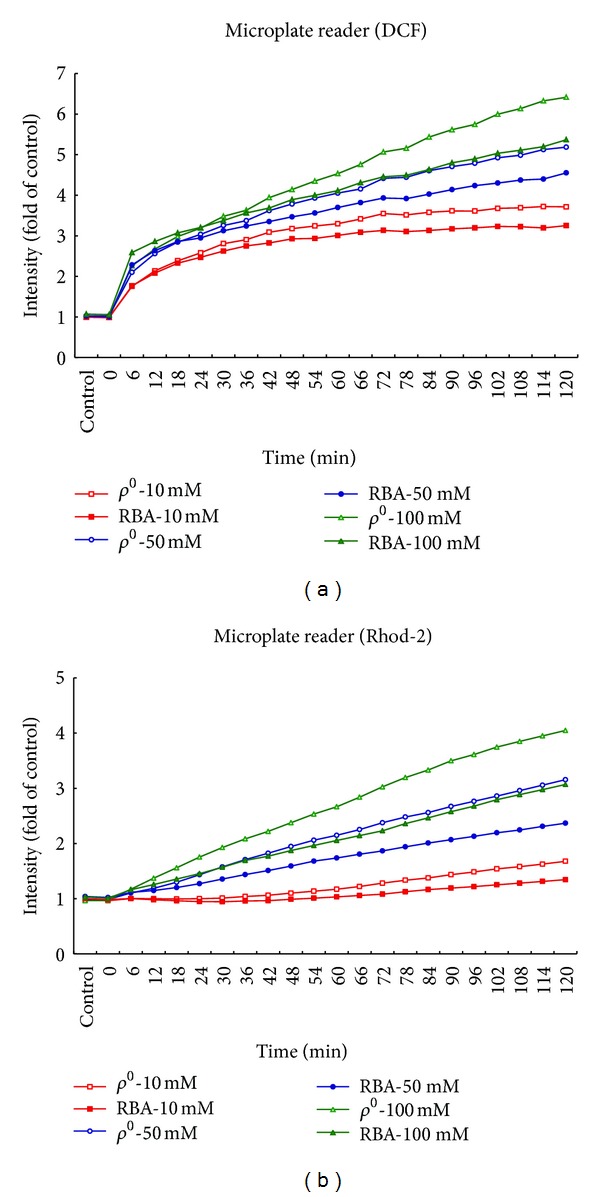
Dual-phase change in mROS and mROS-dependent mCa^2+^ formation during H_2_O_2_-induced oxidative stress in normal and *ρ* ^0^ RBA1 astrocytes. (a) ROS generation detected by DCF. (b) mCa^2+^ detected by Rhod-2. It should be noted that mROS formation and mROS-dependent mCa^2+^concentration in control cells was higher than *ρ* ^0^ astrocytes during the early stage of H_2_O_2_ exposure. However, mitochondrial stress of mROS and mROS-dependent mCa^2+^ stress were significantly higher in *ρ* ^0^ astrocytes than control during long-term H_2_O_2_ exposure.

Next, we investigated whether the resistance to oxidative stress is compromised due to the impact of mitochondrial stress of mROS and mROS-dependent mCa^2+^ stress in *ρ* ^0^ astrocytes. mROS formation and mCa^2+^ concentration were concurrently imaged by dual labeling with a mROS probe, DCF, and Ca^2+^ fluorescent probe, Rhod-2 by laser scanning confocal microscopy. Continuous changes in mitochondrial ROS and Ca^2+^ every 6 min for 2 hr after H_2_O_2_ exposure in both groups were simultaneously analyzed in Figures 5(a) and 5(b), respectively. These results suggest that mROS increased rapidly (within 10 min) after cells were exposed to H_2_O_2_ and this was later accompanied by the increase of mCa^2+^ level. Importantly, mROS formation and mROS-dependent mCa^2+^ concentration in control cells was higher than in *ρ* ^0^ astrocytes during the early stage of 10, 50, and 100 mM H_2_O_2_ exposure. However, mitochondrial stress of mROS and mROS-dependent mCa^2+^ stress were significantly higher in *ρ* ^0^ astrocytes than control during long-term 10, 50, and 100 mM H_2_O_2_ exposure. 

### 3.5. *ρ* ^0^ RBA1 Astrocytes Have Lower DeltaPsi(m) Than Control Cells

**Figure 6:**
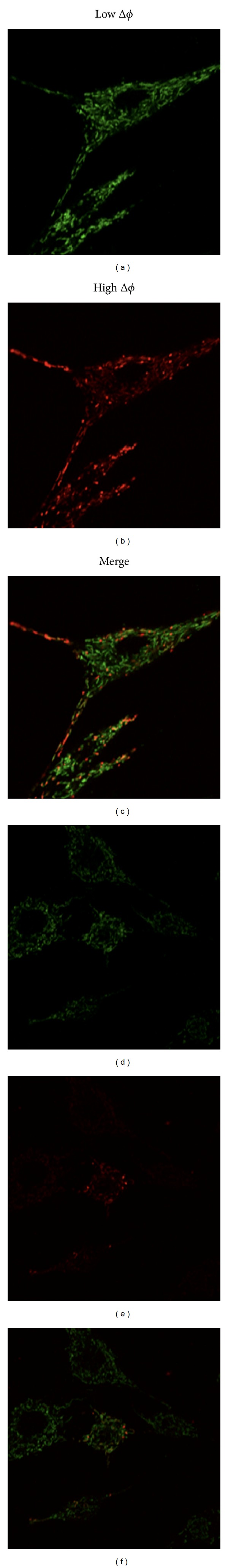
Measurement of DeltaPsi(m) on normal and *ρ* ^0^ RBA1 astrocytes cells by the confocal imaging system. (a–c) Normal RBA1 astrocytes. (d–f) *ρ* ^0^ RBA1 astrocytes. JC-1 measured both high (J-aggregate red fluorescence) (b and e) and low (monomer green fluorescence) (a and d) DeltaPsi(m).

We previously demonstrated that resting mROS level in cells harboring large-scale deletion of mtDNA was lower than that found in cells preserving mtDNA. Besides, resting DeltaPsi(m) in the former cells was found to be less hyperpolarized than that detected in the latter [29]. Moreover, lower DeltaPsi(m) can produce less driving force for Ca^2+^ to enter the mitochondria and consequently reduces mCa^2+^ stress [31]. To investigate whether *ρ* ^0^ astrocytes with lower mROS formation under minor oxidative stress have lower DeltaPsi(m), we detected DeltaPsi(m) changes using a DeltaPsi(m)-sensitive fluorescent probe, JC-1, and evaluated them by laser scanning confocal microscopy. JC-1 measured both high (reported as j-aggregated red fluorescence) and low (reported as monomer green fluorescence) DeltaPsi(m). As indicated in Figure 6, confocal JC-1 imaging demonstrated that DeltaPsi(m) in control cells was heterogeneous (Figures 6(a)–6(c)). Both high (red fluorescence) (Figure 6(b)) and low (green fluorescence) (Figure 6(a)) DeltaPsi(m)s were detected. In comparison, *ρ* ^0^ astrocytes showed that JC-1 expression was much reduced in red (Figure 6(e)) and green (Figure 6(d)) fluorescence. Merge images indicated the decrease of both high and low DeltaPsi(m)s in *ρ* ^0^ astrocytes (Figure 6(c)) when compared to control cells (Figure 6(f)). Taken together, these data provide illustration for the protective effects of *ρ* ^0^ astrocytes via less mROS generation, lower DeltaPsi(m), and therefore lower mCa^2+^ stress. In contrast, *ρ* ^0^ astrocytes render their protection due to augmented mROS and mROS-mediated mCa^2+^ stress under long-term mitochondrial oxidative stress. 

## 4. Discussion

In this study, we demonstrated the biphasic effects of RC defect-augmented mROS-mediated mCa^2+^ stress during H_2_O_2_-induced oxidative damage in *ρ* ^0^ RBA1 astrocytes. We compared the impact of oxidative stress on normal and RC-defective astrocytes during short- and long-term exposure to H_2_O_2_. Our findings indicate that (1) *ρ* ^0^ astrocytes exerted slower O_2_ consumption than control cells, but O_2_ consumption compromised significantly during long-term oxidative stress; (2) cell viability was not different between groups during short-term H_2_O_2_ exposure; (3) cell survival was decreased in *ρ* ^0^ astrocytes incubated with 20 mM H_2_O_2_ during long-term oxidative stress; (4) *ρ* ^0^ astrocytes formed marked mitochondrial swelling, plasma membrane blebs, and earlier positive YO-PRO-1 nuclear staining than control under H_2_O_2_ treatments; and (5) higher mROS formation, mROS-dependent mCa^2+^ level, and DeltaPsi(m) were detected in control cells in early oxidative stress but vice versa during long-term oxidative insult. These findings underscore dual phases of pathological course in RC-defective cells, which has been an area of significant research interest and has emerged as a potential therapeutic target in the treatment of neurological diseases. 

Using time-lapse dual fluorescence imaging microscopy to simultaneously measure the generation of RC defect-enhanced mROS and mCa^2+^, changes of them were visualized using DCF and Rhod-2, respectively. In the current study, we found that mROS generation is prior to mCa^2+^ increase in control and *ρ* ^0^ astrocytes after H_2_O_2_ treatment. These findings were compatible with our previous studies on H_2_O_2_ [32] and photoirradiation-augmented oxidative stress [33–35]. Moreover, our study provided quantitative imaging evidence that dual effect on antioxidation exists in *ρ* ^0^ astrocytes during different extent of oxidative stress. In brief, RC defect protects cells due to less mROS, less DeltaPsi(m), and consequently less mROS-mediated mCa^2+^ stress during short-term oxidative insult. However, such protective effect obtained from *ρ* ^0^ cells does not last long due to significantly compromised RC activity indicated from O_2_ consumption, significantly augmented mROS-enhanced mitochondrial stress, and mROS-dependent mCa^2+^ stress during long-term oxidative stress.

The paradoxical nature of mROS has been described as a double-edged sword: they protect at basal level but may also damage at excessive level [36]. Similarly, moderate increase in mCa^2+^ is physiologically relevant, but Ca^2+^ overload is detrimental to mitochondrial function. The crosstalk between Ca^2+^ and ROS signaling systems is critical to maintain physiological homeostasis. Minor mROS formation can reduce mCa^2+^ level to exert protective preconditioning [37]. In contrast, severe mROS formation can enhance mCa^2^increase. The combined effect of mCa^2+^ and mROS may facilitate the opening of the MPT pore [38]. The opening of the MPT pore leads to DeltaPsi(m) depolarization and results in mitochondrial swelling. The outer mitochondrial membrane ruptures due to mitochondrial swelling result in the release of cytochrome c, leading to the inhibition of electron transport and enhancing more ROS production [15]. Thus, oxidative stress resulted from such feed-forward loops could be devastating creating cellular damage far beyond direct Ca^ 2+^-induced damage. 

Controversy exists as to whether *ρ* ^0^ cells are resistant to oxidative stress or not. The effects of these RC-defective cells are diverse, and both detrimental and protective to cell survival have been described. Cells-harboring mtDNA mutations have been shown to induce protective expression of Bcl-2 and Bfl-1, prosurvival proteins [39]. It has been reported that DeltaPsi(m) in *ρ* ^0^ cells might be distinctly lower than that of wild-type cells [40]. In contrast, we previously showed that common deletion- (CD-) induced RC defect results in significantly elevated mROS and depolarized DeltaPsi(m) and can effectively lead to an enhanced apoptotic signaling [29]. As the dual effect of mROS, whether it is protective or detrimental depends on the produced amount. Similarly, whether *ρ* ^0^ cells exert protection or damage to cell survival depends on the extent of oxidative stress and consequent mROS production. Future studies are warranted to examine this putative mechanism in *ρ* ^0^ cells.

In conclusion, we demonstrated dual phases of RC defect-augmented mROS-mediated mCa^2+^ stress during oxidative insult in a cell culture model of *ρ* ^0^ astrocytes. RC defect protects cells due to less mROS and mROS-dependent mCa^2+^stress in minor oxidative insult. RC-defect-mediated protection is compromised due to significantly augmented mROS and mROS-dependent mCa^2+^stress during long-term oxidative stress. The key point that *ρ* ^0^ astrocytes have lower DeltaPsi(m) than normal RBA1 underscore the dual phases of modulation for cell survival and cell death. 

## Figures and Tables

**Table 1:** Different effects of RC-augmented mitochondrial stress following H_2_O_2_ treatment on mitochondrial swelling, plasma membrane blebbing, YO-PRO-1 stain, and nuclear condensation in normal and *ρ* ^0^ RBA1 astrocytes.

Cell line	Mitochondria swelling	Blebbing	1/2 cells Yo-Pro	Cells Yo-Pro	Condensation of nucleus (%)
RBA-1	20	70	140	150	7.2%
*ρ* ^0^ (RBA-1)	10	70	120	130	8.9%
